# Aliskiren Attenuates the Inflammatory Response and Wound Healing Process in Diabetic Mice With Periodontal Disease

**DOI:** 10.3389/fphar.2019.00708

**Published:** 2019-07-04

**Authors:** Sandra Helena Penha Oliveira, Victor Gustavo Balera Brito, Sabrina Cruz Tfaile Frasnelli, Bianca da Silva Ribeiro, Milena Nunes Ferreira, Dayane Priscilla Queiroz, Carluci Taís Beltan, Vanessa Soares Lara, Carlos Ferreira Santos

**Affiliations:** ^1^Department of Basic Sciences, School of Dentistry of Araçatuba, São Paulo State University (UNESP), São Paulo, Brazil; ^2^Programa Multicêntrico de Pós-graduação em Ciências Fisiológicas, School of Dentistry of Araçatuba, São Paulo State University (UNESP), São Paulo, Brazil; ^3^Department of Stomatology, Bauru School of Dentistry, University of São Paulo (USP), Bauru, Brazil; ^4^Department of Biological Sciences, Bauru School of Dentistry, University of São Paulo (USP), Bauru, Brazil

**Keywords:** renin–angiotensin system, diabetes, periodontal disease, inflammation, renin, collagen, cytokine, chemokines

## Abstract

The aim of this study was to characterize the role of local RAS (renin–angiotensin system) in the inflammatory response of normal (N) and diabetic (D) mice with periodontal disease (PD). Diabetes Mellitus (DM) was induced by peritoneal injection of streptozotocin in Balb/c mice. PD was induced by ligature around the first molar in both N and D, irrespective of whether they were treated with aliskiren (50 mg/kg, Alisk). Mandibles were harvested for histomorphometric analyses, and gingival tissue (GT) was collected to evaluate gene expression and extracellular matrix components (ECM). Immunohistochemical (IHC) analyses were used to localize RAS in GT. The production of C-reactive protein (CRP), IL-1β, CXCL2, and CCL8 was evaluated by enzyme-linked immunosorbent assay (ELISA). Renin was found to exacerbate the inflammation and periodontal bone loss at 14 days after PD, and Alisk inhibited this process in GT of N and D. PD increased CRP, CXCL2, CCL8, and IL-1β production in both animals. Alisk could inhibit CRP, CXCL2, and CCL8 primarily in D animals. However, only CCL8 was decreased in N animals after Alisk pretreatment. PD enhanced expression and production of AGT, ACE, AT1R, and AT2R in both N and D. AT1R expression was higher in D with PD, and AT2R expression was higher in N with PD. ACE2 and receptor Mas (MasR) expression and production was elevated in the control group of both animals. PD inhibited ACE2 in N but not in D. MasR expression was unaffected in both N and D with PD. Alisk reduced expression and production of all RAS components in GT of both animals, except for ACE2 in N. RAS staining was observed in all layers of epithelium, basal cell layer, and lamina propria and was higher in N with PD. Col1a1, Col1a2, Col3a1, and fibronectin (Fn1) were increased in both animals with PD. Alisk inhibited Col1a1 and Fn in both animals, Col1a2 was decreased only in D, while levels of Col3a1 remained unchanged in all animal groups. In conclusion, these data demonstrated the presence and functional role of local RAS in GT, exacerbating the inflammatory response, periodontal bone loss, and wound healing processes in both N and D animal groups. In addition, Alisk was able to significantly reduce gingival inflammation, excessive wound healing processes, and periodontal bone loss.

## Introduction

Systemic renin–angiotensin system (RAS) is an endocrine axis responsible for modulating a variety of physiological and pathological processes such as blood pressure, hydroelectrolytic balance, inflammation, and oxidative stress (Zablocki and Sadoshima, 2013; Itinteang et al., 2015; Wang et al., 2015; Xue et al., 2016). The classical RAS consists of circulating renin, acting on angiotensinogen (AGT) to produce angiotensin I (AngI), which in turn is converted into angiotensin II (AngII) by angiotensin-converting enzyme (ACE). Some studies have demonstrated the presence of local RAS components in oral tissues such as guinea pig and rabbit gingival fibroblasts (Ohuchi et al., 2002; Ohuchi et al., 2004) and ferret gingiva *in vivo* (Berggreen and Heyeraas, 2003). Recent studies have demonstrated an association between diabetes and RAS, particularly a prominent involvement of AngII in diabetic complications, such as nephropathy and cardiovascular dysfunction (Ribeiro-Oliveira et al., 2008). One of the most important landmarks was the discovery of local RAS as described by Santos et al. (2009), where the existence of local RAS in rat gingival tissue was demonstrated for the first time. These authors reported that antagonizing AT1R and renin can significantly prevent periodontal bone loss induced by PD in rats (Santos et al., 2015). Moreover, recent studies have shown that RAS blockers are able to reduce the inflammation and, therefore, decrease the expression of matrix metalloproteinases (MMPs) in RANK/RANKL axis and cathepsin K in a rat model of PD (Araújo et al., 2013). Furthermore, our knowledge about this concept is growing due to increase in the number of studies that have described treatment of mice with ACE inhibitor preventing bone loss Gonçalves-Zillo et al. (2013), the protective effect of ACE inhibitors in PD-induced and arthritis-associated alveolar bone loss (Queiroz-Junior et al., 2015), and the mechanism by which AT1R inhibitor prevents inflammation and alveolar bone loss in periodontitis (Li et al., 2018).

Periodontal disease (PD) has a high prevalence worldwide and is considered to be the second largest cause of dental pathology in human population (Mumghamba et al., 1995). It is characterized by a chronic infection leading to destruction of periodontal tissues, resulting in loss of connective soft tissue and bone and formation of periodontal pockets that eventually cause teeth loss (Okada and Murakami, 1998; Bascones-Martinez et al., 2011). Most of these disease pathologies are caused due to an exacerbated inappropriate immune response. The circulating cytokines and chemokines have been detected at elevated levels in the gingival crevicular fluid (GCF) and saliva of patients who have PD, making them putative biomarkers of the disease (Lamster, 1997; Gemmell et al., 2001; Sexton et al., 2011; Stadler et al., 2016; Aldahlawi and Youssef, 2018). CXCL2-like-related chemokines are powerful neutrophil chemoattractants and are involved in many immune responses including wound healing and periodontitis. Elevated levels of CXCL2 leads to increased recruitment of neutrophils to periodontal ligaments in diabetic mice with induced PD (Greer et al., 2016; Zheng et al., 2018). Another important chemokine is CCL8, which is involved in leukocyte cell activation and is associated with the severity of periodontitis (Panezai et al., 2017). The association of periodontitis with systemic inflammation diseases has been a target of various studies to understand the mechanisms involved in the process.

Diabetes has been confirmed as a major risk factor for periodontitis (Khader et al., 2006; Chávarry et al., 2009). Diabetic patients have impaired immune functioning, especially due to polymorphonuclear cells, which when present reduce phagocytic and microbicidal functions in the patient’s body (Drachman et al., 1966). The decrease in cellular functions occurring in diabetes is due to high concentrations of glucose to which the host cells are exposed, reducing immunological function and making the host more susceptible to develop a variety of pathologies including periodontitis (Loe, 1993; Marhoffer et al., 1992; Geerlings and Hoepelman, 1999; Shah and Hux, 2003; Muller et al., 2005). In our study, we used a direct renin inhibitor available for clinical use. This pharmacological modulation may be a useful tool to understand the effects of this drug in the interaction between RAS, PD, and systemic disease, thereby improving the quality of life of individuals with these pathologies. Thus, the aim of this study was to characterize the local RAS and the role of renin in inflammatory response of normal (N) and diabetic mice (D) after experimental PD was induced. We observed that renin exacerbates the inflammatory response and periodontal bone loss in PD-induced mice, and this effect is more intense in hyperglycemic animals. Furthermore, we verified the existence of the components of local RAS in the gingival tissue as well as their role in tissue repair.

## Materials and methods

### Animals, Ethical Aspects, and Experimental Design

Animal care and experimental protocols were in accordance with the Brazil’s National Council for Animal Experiments Control and were approved by the Ethics Committee on Animal Use from School of Dentistry of Araçatuba (UNESP) (Process FOA-00106-2016). Efforts were made to minimize animal suffering and reduce the number of subjects. Thirty six adult male Balb/c mice (30 ± 5 g) from the Animal Facility of the Department of Basic Sciences (School of Dentistry of Araçatuba, UNESP) were used. Animals were housed in individually ventilated cages in an environment with controlled light (12 h light/dark cycle), temperature (22 ± 2°C), and humidity (55 ± 5%), offered with standard pellet diet and drinking water ad libitum. The animals were randomly divided into six experimental groups (n = 12), as follows: Normal Control, Normal with PD, aliskiren-treated normal animals with PD, Diabetic Control, Diabetic with PD, and aliskiren-treated diabetic animals with PD.

### Diabetes Induction

For diabetes induction, mice received 200 mg/kg streptozotocin intraperitoneally (Sigma-Aldrich, St. Louis, MO, USA) diluted in citrate buffer (100 mM, pH 4.5). After 7 days, glycemia levels were measured by a glucometer (One Touch Ultra 2, Johnson & Johnson Medical, Milpitas, CA, USA) and animals were considered to be diabetic when values are shown to be 250 mg/dl or higher (Kim et al., 2014).

### Periodontal Disease Induction

PD was induced by bilateral insertion of a ligature around the lower first molar, kept for 14 days, as previously described (Bonato et al., 2012). Briefly, the animals were anesthetized with 80 mg/kg ketamine hydrochloride (Cetamim, Syntec; Hortolândia, São Paulo, Brazil) associated with 10 mg/kg xylazine hydrochloride (Calmium, AgenerUnião; Embu-Guaçu, São Paulo, Brazil) and were placed in the ventral decubitus position at a dental table for rodents, with oral retractors supported on the incisive teeth. A 4-0 silk thread (Shalon; Goiânia, Goiás, Brazil) was wrapped around the first inferior molars, carefully pushing the ligature into the gingival sulcus, and knotting mesially. Control groups did not receive the ligature. However, they were anesthetized and manipulated similarly to undergo the same stress as the others.

### Aliskiren Treatment

Aliskiren was daily administered, at 50 mg/kg on PBS pH 7.4, as described by Santos et al. (2015) using an oral gavage, starting 1 day before PD induction and maintained until euthanasia. Control groups were manipulated similarly and received only PBS pH 7.4 to undergo the same stress as the others.

### Euthanasia and Sample Harvest

On day 15 after PD induction, the animals were euthanized by halothane (Tanohalo; Cristália, Itapira, SP, Brazil) by overdose inhalation. The presence of bilateral ligature was evaluated, and the animals in which it was absent were excluded from the study. The marginal gingival was surgically harvested in clean condition, immediately frozen in liquid nitrogen, subsequently stored at −80°C until used for cytokine quantification and gene expression analyses, or fixed for histological processing. Hemi-mandibles were also harvested to evaluate the alveolar bone lost by histological methods.

### Histomorphological Analysis

Gingival tissue and hemi-mandible were fixed in 10% formaldehyde buffered solution for 48 h. Tissue was washed under tap water for 24 h, and hemi-mandibles were decalcified with 10% ethylenediaminetetraacetic acid solution for 60 days. After this, tissue was dehydrated and embedded in paraffin and 5-µm-thick sections longitudinally were obtained. To determine bone resorption, the total area of furcation region and the alveolar bone area within were measured with the software ImageJ (Version 1.47, National Institutes of Health, Bethesda, MD, USA). Finally, the alveolar bone percentage was calculated.

### Immunohistochemical Analysis

Paraffin sections were obtained as described above and immunostained to determine the expression of renin (Renin), angiotensinogen (AGT), angiotensin I converting enzyme 1 (ACE), angiotensin I converting enzyme 2 (ACE2), angiotensin II receptor type 1 (AT1R), angiotensin II receptor type 2 (AT2R), and receptor Ag1-7 Mas (MasR). Primary antibodies that were used (Santa Cruz Biotechnology, Santa Cruz, CA, USA) included rabbit polyclonal anti-renin (1:50), rabbit polyclonal anti-AGT (1:50), rabbit polyclonal anti-ACE (1:50), anti-ACE2 (1:50), goat polyclonal anti-AT1R (1:250), anti-AT2R (1:100), and goat polyclonal anti-MasR (1:75). Antibodies binging was detected using Histofine^®^ Simple Stain™ Mouse MAX PO (goat and rabbit) (Nichirei Biosciences Inc.; Tokyo, Japan) and 3,3’-diaminobenzidine-tetrahydrochloride (Dako Corp., Carpinteria, CA, USA) as chromogenic substrate. The procedure was completed using Harry’s hematoxylin for counterstaining. All assays were accompanied by a negative control where primary antibody was not used. Positive controls were performed on paraffin-embedded longitudinal sections of kidney for Renin, ACE, ACE2, AT1R, AT2R, and MasR and longitudinal sections of heart for AGT. For immunohistochemical (IHC) analysis, five sections from each group were randomly selected to examine the immunolabeling in gingival epithelium and connective tissue and were performed by three independent and blind researchers. In brief, expression was based on intensity of the immunostaining [negative (−); weak (+); moderate (++); strong (+++)], as described by Santos et al. (2015) with some modifications. Representative slides were photographed with a digital camera (Olympus, XC50, Tokyo, Japan), which was coupled to a light microscope (Olympus, BX53, Tokyo, Japan).

### Cytokines Quantification by Enzyme-Linked Immunosorbent Assay

The gingival samples from the same animal were pooled together and sonicated until total tissue was disintegrated in lysis buffer (PBS pH 7.4 plus protease inhibitor cocktail), which was then centrifuged, and the supernatant was used for quantification of C-reactive protein (CRP), IL-1β, CXCL2, and CCL8 by sandwich enzyme-linked immunosorbent assay (ELISA). Each pool was considered n = 1, and final experimental number was n = 5. Antibodies and standards were purchased from R&D Systems (Minneapolis, MN, USA), and antibody concentrations that were used had been previously standardized. Assays were performed in 96-well microplates coated with the respective capture antibodies for 18 h at 4°C. Nonspecific binding sites were blocked with blocking buffer (PBS plus 1% bovine serum albumin) for 2 h at room temperature. Samples and standards were loaded and incubated for 2 h at room temperature under constant agitation. Respective biotinylated antibody was added and incubated for 1 h at room temperature. Horseradish peroxidase-conjugated streptavidin in blocking buffer was added and incubated for 1 h at room temperature, followed by addition of chromogenic substrate solution (3,3′,5,5′-tetramethylbenzidine). Reaction was conducted for 30 min and stopped with 4 M sulfuric acid, and OD was measured at wavelength 450 nm. Plates were washed four times with washing buffer (PBS pH 7.4, with 0.1% Tween 20) after each step. Standard curve consisted of two-fold serial dilution of respective recombinant cytokines in blocking buffer (ranging from 2000 to 1.95 pg/ml), and their concentrations in samples were calculated in relation to the standard curve.

### Gene Expression Analysis by Real-Time Reverse Transcription Polymerase Chain Reaction

Gingival tissue was homogenized with TRIzol reagent (Invitrogen, Thermo Fisher Scientific; Carlsbad, CA, USA), and total RNA was extracted following the manufacturer’s instructions. Samples were treated with DNAse I (Sigma-Aldrich), RNA was quantified by fluorometry with commercial kit (Quant-iTRiboGreen RNA Assay Kit, Invitrogen), and samples were reverse transcribed to complementary DNA with High Capacity RNA-to-cDNA™ Kit (Applied Biosystems, Thermo Fisher Scientific; Foster City, California, USA), according to the manufacturer’s instructions.

Gene expression analysis of RAS and tissue repair targets was performed by real-time reverse transcription polymerase chain reaction (qRT-PCR), using StepOnePlus™ Real-Time PCR Systems and TaqMan™ Gene Expression Assays (FAM fluorophore reporter/nonfluorescent quencher MGB) (Applied Biosystems, Thermo Fisher Scientific), and assay references are listed in the following table. The relative amount of transcripts was determined by the 2−(∆∆CT) method, with target expression normalization done with glyceraldehyde-3-phosphate dehydrogenase as housekeeping gene, and normal control NC as the calibrator.

**Table:** 

RAS components			Accession number (RefSeq)
Renin	Renin	Mm02342887_mH	NM_031192.3
Agt	Angiotensinogen	Mm00599662_m1	NM_007428.3
ACE	Angiotensin I converting enzyme 1	Mm00802048_m1	NM_001281819.1
ACE2	Angiotensin I converting enzyme 2,	Mm01159006_m1	NM_001130513.1
AT1R	Angiotensin II receptor type 1a	Mm01957722_s1	NM_177322.3
AT2R	Angiotensin II receptor type 2,	Mm00431727_g1	NM_007429.5
Mas1	MAS receptor	Mm00434823_s1	NM_008552.4
Tissue repair			
Col1a1	Collagen type I alpha 1	Mm00801666_g1	NM_007742.3
Col1a2	Collagen type I alpha 2,	Mm00483888_m1	NM_007743.2
Col3a1	Collagen type III alpha 1,	Mm00802300_m1	NM_009930.2
Fn1	Fibronectin 1	Mm01256744_m1	NM_001276408.1
Housekeeping gene			
Gapdh	glyceraldehyde-3-phosphate dehydrogenase	Mm99999915_g1	NM_001289726.1

### Statistical Analysis

After passing the Shapiro–Wilk normality distribution test, data were analyzed by one-way ANOVA, followed by Sidak *post hoc* test. The data are represented as column graphs, plotted with mean and standard error. Non-parametric data (IHC analysis) are represented as a box plot graph, plotted with median and minimum and maximum values. Such data were analyzed by non-parametric Kruskal–Wallis test followed by the Dunn’s *post hoc* test. For all analyses, p < 0.05 was considered statistically significant and multiple comparisons comprised of (a) Control vs. PD, (b) PD vs. Alisk-treated animals with PD, and (c) Normal vs. Diabetic in the same experimental condition. All analysis was carried out in the statistical software Graph Pad Prism v7.0 (GraphPad Software Inc.; San Diego, California, USA).

## Results

### Renin Is Related to PD-Induced Periodontal Bone Loss and Inflammatory Response in a Diabetic Mouse

**Figure 1:**
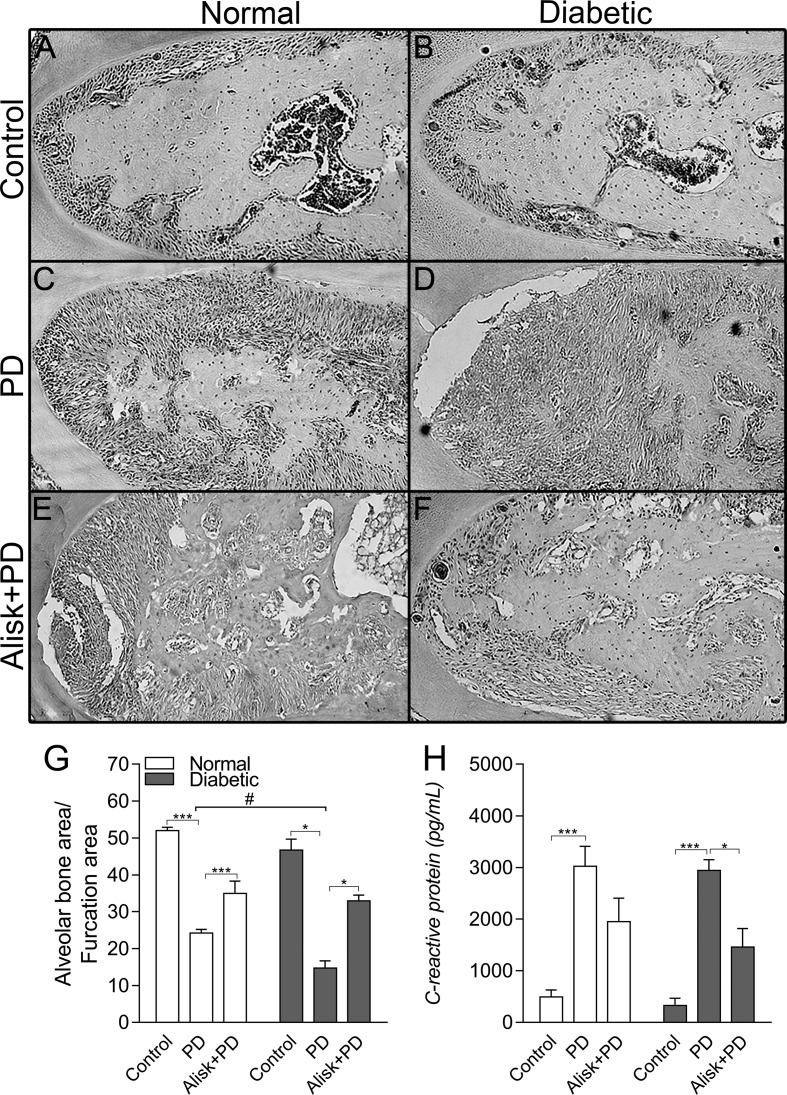
Renin contributes to the periodontal bone loss and inflammation response in normal and diabetic mice 14 days after PD. Histological sections of the lower first molars from the normal mice [control **(A)**, normal with PD **(C)**, normal with PD treated with aliskiren (Alisk) **(E)**], diabetic mice [control **(B)**, diabetic with PD **(D)**, and diabetic with PD and treated with Alisk **(F)**]. Percentage of alveolar bone area per μm^2^ computed using Image J **(G)** software. C-reactive protein production by gingival fibroblast in normal and diabetic mice 14 days after PD **(H)**. Data are represented as mean ± SEM (n = 10). Significant values are represented as ***p < 0.001 and *p < 0.05 (Control vs. PD; PD vs. Alisk + PD; and PD vs. PD+Alisk), and ^#^p < 0.05 (diabetic + PD vs normal + PD).

Firstly, we assessed the PD-induced bone loss in the first molar furcation region by histomorphometry on HE-stained mandible sections. Groups with PD had significant bone loss compared to Control. As expected diabetic mice presented more severe periodontal destruction compared to normal mice with PD (**Figure 1C, D, and G**). Treatment with aliskiren (50 mg/kg, Alisk) was able to significantly reduce periodontal bone loss, in both conditions of normal and diabetic with PD, as compared to non-treated mice (**Figure 1C** vs **1E**, and **Figure 1D** vs **1F**).

Inflammatory response was evaluated by gingival CRP production by ELISA, which showed to be elevated in both normal and diabetic PD groups. Alisk treatment could significantly reduce it only in diabetic animals group (**Figure 1H**).

### Renin Modulates CXCL2 and CCL8 Production Induced by PD on Gingival Tissue in Diabetic Mice

**Figure 2:**
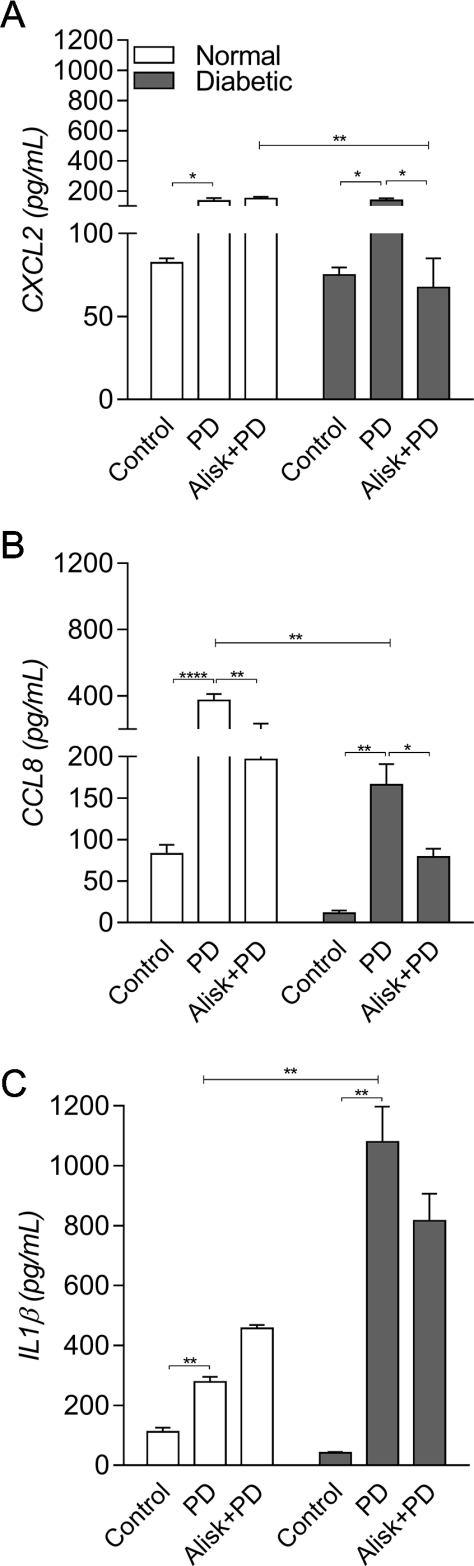
Production of CXCL2, CCL8, and IL-1β by gingival tissue from normal and diabetic mice 14 days after PD. **(A)** CXCL2, **(B)** CCL8, and **(C)** IL1β by ELISA. Data represented as mean ± SEM (n = 10). Statistical differences represented by *p < 0.05, **p < 0.01, and ****p < 0.0001 (Control vs. PD; PD vs. Alisk + PD; and PD vs. PD+Alisk).

Next, we evaluated IL-1β and chemokine production by ELISA in gingival tissue from normal and diabetic mice with PD for both treated and not treated with Alisk groups. PD caused an increase in CXCL2 and CCL8, in both animals, compared to its respective control. Alisk treatment significantly reduced CCL8 production but not CXCL2 in normal mice, while in diabetic mice Alisk was able to significantly reduce both CXCL2 and CCL8 production (**Figure 2A and B**). Interleukin-1β production increased after PD induction, but Alisk did not modify the effect in either group of animals (**Figure 2C**).

### PD-Induced RAS Component Expression May be Modulated by Renin

With an aim to investigate whether the RAS components are produced in local gingival tissue and are involved in the inflammatory process in the PD-induced events, we next evaluated the gene expression of *Renin*, *AGT*, *ACE*, *AT1R*, *AT2R*, *ACE2*, and *MasR* by qRT-PCR in gingival tissue from normal and diabetic mice with PD for both groups in which mice were treated or not treated with Alisk.

**Figure 3:**
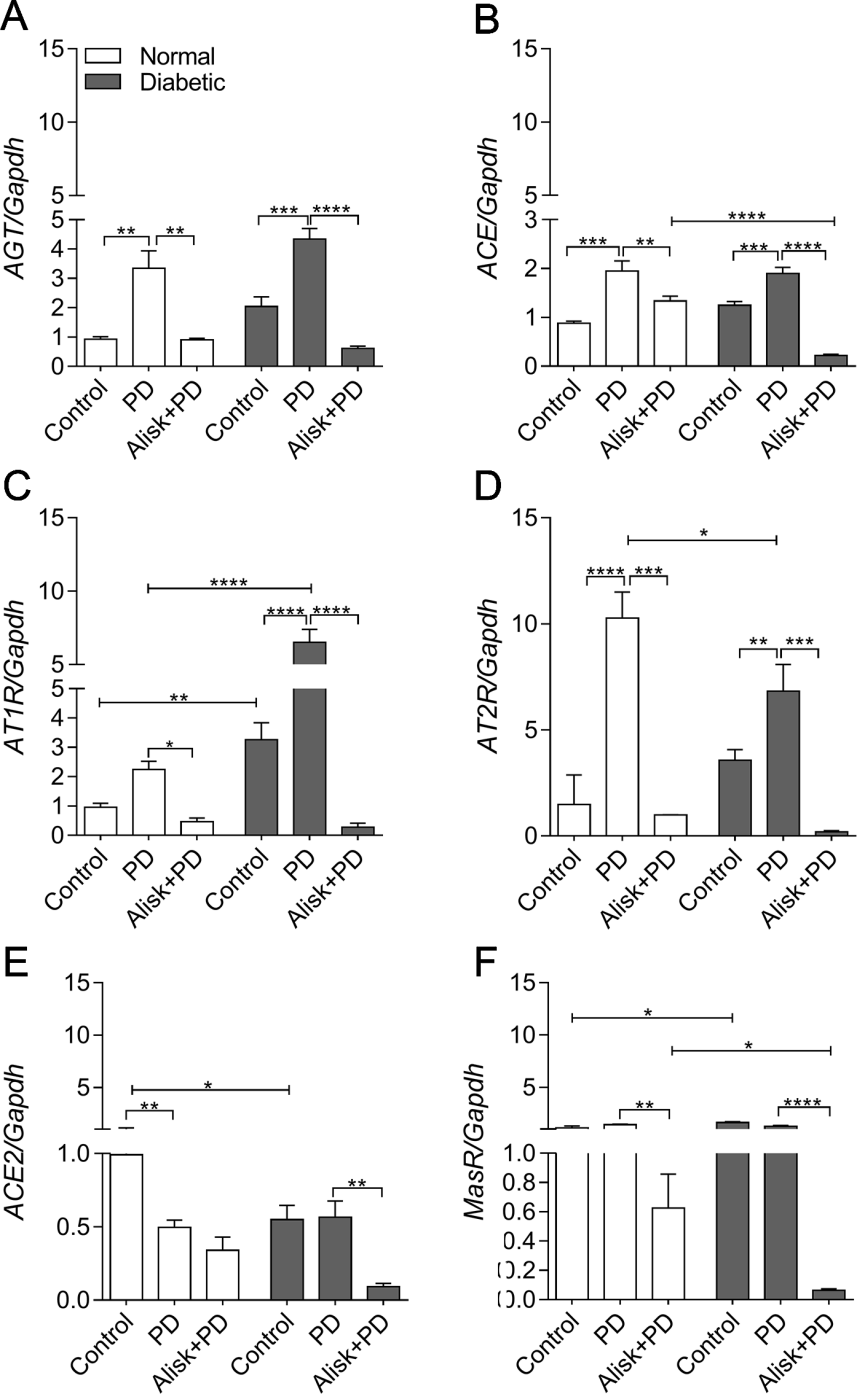
*AGT*, *ACE*, *AT1R*, *AT2R*, *ACE2*, and *MasR* gene expression of gingival tissue from normal and diabetic mice 14 days after PD. **(A)** *AGT*, **(B)** *ACE*, **(C)** *AT1R*, **(D)** *AT2R*, **(E)** *ACE2*, and **(F)** *MasR* in gingival tissue of control, normal mice with PD (PD), normal mice with PD treated with aliskiren (Alisk + PD), control, diabetic mice with PD (PD), diabetic mice with PD treated with aliskiren (Alisk + PD) by real-time RT-PCR. Results express mean ± SEM, normalized by fold change. Significant values are represented by *p < 0.001, **p < 0.05, ***p < 0.005, and ****p < 0.0001 (Control vs. PD, PD vs. Alisk+ PD, PD vs. PD, PD+Alisk vs. PD+Alisk).

First of all, we observed that *renin* expression was undetectable in the gingival tissue by qRT-PCR (data not shown). PD increased *AGT* and *ACE* expression in both normal and diabetic mice, compared to control. Alisk treatment significantly decreased *AGT* expression in both conditions, while *ACE* expression was decreased only in diabetic treated animals (**Figure 3A and B**). Angiotensin II (AngII) receptors, *AT1R* and *AT2R*, are expressed in greater amount in diabetic animal, as compared to normal animal. PD did not alter *AT1R* expression in normal mice but it was significantly increased in diabetic mice. Alisk treatment significantly reduced its expression in normal mice and significantly in diabetic mice. PD increased *AT2R* expression but more pronouncedly in normal than in diabetic mice, while Alisk treatment decreased this response in both normal and diabetic mice (**Figure 3C and D**).

Animals in both control and PD groups showed increased expression of *ACE2*, but it remained unaltered in diabetic group. Alisk significantly reduced its expression only in diabetic mice (**Figure 3E**). Angiotensin 1–7 receptor, *MasR*, was also evaluated, and it was observed to have higher expression in control animals. PD was able to reduce the expression only in diabetic mice, and Alisk treatment reduced the expression in both animals, but significantly in diabetic mice (**Figure 3F**).

### Renin Inhibition Reduces the Production of RAS Components in Gingival Tissue

To confirm the gene expression profile, we analyzed the presence and localization of proteins of RAS components by IHC in gingival tissue in the proposed experimental conditions. Furthermore, we used pharmacological agents to block the renin component. Our results demonstrated positive immunolabeling for Renin, AGT, ACE, ACE2, AT1R, AT2R, and MasR, confirming the presence of local RAS in gingival tissue; however, the immunolabeling profile of each target was different.

**Figure 4:**
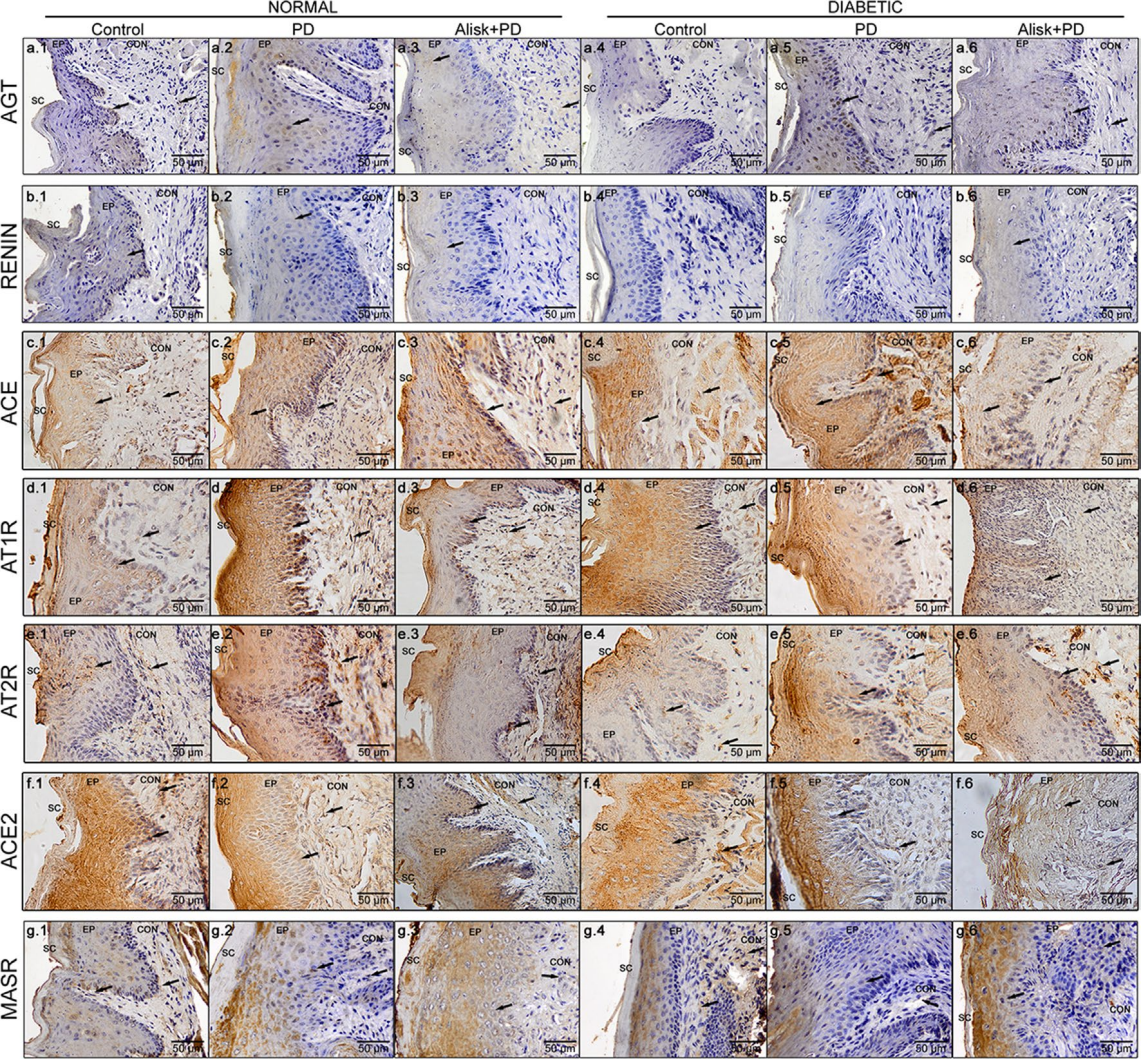
Immunoreactivity for AGT, renin, ACE, AT1R, AT2R, ACE2, and MasR in gingival tissue of normal and diabetic mice 14 days after PD. AGT production of normal mice (a.1 control, a.2 with PD, and a.3 with Alisk + PD) and diabetic mice (a.4 control, a.5 with PD, and a.6 with Alisk + PD); Renin production of normal mice (b.1 control, b.2 with PD, and b.3 with Alisk +PD), and diabetic mice (b.4 control, b.5 with PD, and b.6 with Alisk+PD); ACE production of normal mice (c.1 control, c.2 with PD, and c.3 with Alisk + PD), and diabetic mice (c.4 control, c.5 with PD, and c.6 with Alisk + PD); AT1R production of normal mice (d.1 control, d.2 with PD, and d.3 with Alisk +PD), and diabetic mice (d.4 control, d.5 with PD, and d.6 with Alisk+PD); AT2R production of normal mice (e.1 control, e.2 with PD, and e.3 with Alisk + PD), and diabetic mice (e.4 control, e.5 with PD, and e.6 with Alisk + PD); ACE2 production of normal mice (f.1 control, f.2 with PD, and f.3 with Alisk +PD), and diabetic mice (f.4 control, f.5 with PD, and f.6 with Alisk+PD); MasR production of normal mice (g.1 control, g.2 with PD, and g.3 with Alisk +PD), and diabetic mice (g.4 control, g.5 with PD, and g.6 with Alisk+PD). All images are at 20× magnification, and scale bars indicate a distance of 50 µm. Brow staining indicates positive IR. Abbreviations: sc, superficial cells; ep, epithelium; BL, basal cell layer; LP, lamina propria.

In normal mice, the IR for AGT was found moderate in the epithelium and basal cell layer of gingival in control animals, but weak in the lamina propria (**Figure 4a.1**). In PD-induced animals, we observed moderate IR in the gingival epithelium, weak IR in basal cell layer, and negative IR in lamina propria of the gingival (**Figure 4a.2**). In PD animals treated with Alisk group, the IR was negative in the epithelium and basal cell layer of gingival and weak in the lamina propria (**Figure 4a.3**). In diabetic animals, the IR for AGT was absent in the control group but was strong in PD animals and moderate in PD animals treated with Alisk (**Figure 4a.4, a.5, and a.6**). In control group of normal mice, IR for rennin was weak in gingival epithelium, basal cell layer, and lamina propria (**Figure 4b.1**). Induction of PD and Alisk treatment did not induce any alteration in the IR (**Figure 4b.2 and b.3**). In diabetic mice, we did not find any immunostaining for renin in any part of the gingival tissue (**Figure 4b.4, b.5, and b.6**). Weak IR for ACE was found in the epithelium and basal cell layer of the gingival tissue but was negative in the lamina propria in the control group of normal mice (**Figure 4c.1**). In PD group, the IR for ACE was moderate to strong in the epithelium, basal cell layer, and lamina propria. Alisk did not produce any changes in the IR in gingival tissue when compared to PD group (**Figure 4c.2 and c.3**). In diabetic mice, the immunostaining in the gingival epithelium of the control group was negative, in the basal cell layer was weak, and in the lamina propria was moderate (**Figure 4c.4**). In PD group, IR was negative in the gingival epithelium and moderate in the basal cell layer and lamina propria of the gingival tissue (**Figure 4c.5**). Pre-treatment with Alisk induced a weak IR in the gingival epithelium and was able to inhibit the IR for ACE in the basal cell layer and lamina propria of the gingival tissue (**Figure 4c.6**). IR for AT1R was found to be weak in the epithelium, basal cell layer, and lamina propria of the gingival tissue in normal mice (**Figure 4d.1**). In PD group, IR was negative in the epithelium, strong in the basal cell layer, and moderate in the lamina propria (**Figure 4d.2**). Alisk was able to reverse IR in the epithelium and decrease the IR in the basal cell layer and lamina propria of the gingival tissue (**Figure 4d.3**). In diabetic mice, AT1R IR was weak in the epithelium and basal cell layer and moderate in the lamina propria of gingival tissue in the control group (**Figure 4d.4**). In PD group, IR was negative in the epithelium, moderate in the basal cell layer, and decreased in the lamina propria of the gingival tissue (**Figure 4d.5**). Alisk increased the immunostaining for AT1R in the epithelium, decreased in the basal cell layer, and maintained in the lamina propria (**Figure 4d.6**). AT2R IR was found to be moderate in the epithelium, basal cell layer, and lamina propria in the control group of normal mice (**Figure 4e.1**). In PD group, IR was observed to be weak in the epithelium and strong in the basal cell layer and lamina propria of the gingival tissue (**Figure 4e.2**). Alisk inhibited IR in the epithelium and basal cell layer and maintained in the lamina propria of the gingival tissue (**Figure 4e.3**). In diabetic mice, IR for AT2R was weak in epithelium, basal cell layer, and lamina propria in the control group in the normal mice (**Figure 4e.4**). In PD group, IR was increased since it was negative in the epithelium, weak in the basal cell layer, and moderate in the lamina propria of the gingival epithelium (**Figure 4e.5**). In the presence of Alisk, the IR for PD group did not change in the whole gingival tissue (**Figure 4e.6**). For ACE2, IR was found negative in the epithelium and moderate in the basal cell layers and lamina propria of the gingival tissue in the normal mice (**Figure 4f.1**). PD increased IR in the epithelium, basal cell layer, and lamina propria (**Figure 4f.2**). Pre-treatment of the Alisk did not modify IR in the whole gingival tissue (**Figure 4f.3**). In diabetic mice, IR was observed to be negative in the epithelium and weak in the basal cell layer and lamina propria of the gingival tissue (**Figure 4f.4**). Immunostaining for PD group was weak in the epithelium and moderate in the basal cell layer and lamina propria (**Figure 4f.5**). Alisk treatment was able to inhibit the IR in the whole gingival tissue in these mice (**Figure 4f.6**). In relation to MasR, IR was not found in the epithelium and was weak in the basal cell layer and lamina propria of the gingival tissue in the control group of normal mice (**Figure 4g.1**). PD increased the IR in the epithelium but maintained in the basal cell layer and lamina propria (**Figure 4g.2**). Addition of Alisk inhibited the IR in the epithelium but did not change it in the basal cell layer and lamina propria of the gingival tissue of normal mice (**Figure 4g.3**). In diabetic mice, IR for MasR was negative in the epithelium and weak in the basal cell layer and lamina propria of the gingival tissue (**Figure 4g.4**). PD increased the IR in the epithelium but not in the basal cell layer and lamina propria (**Figure 4g.5**). Alisk did not alter the IR for MasR in whole gingival tissue (**Figure 4g.6**).

### Renin Modulates *Col1a1*, *Col1a2*, and *Fibronectin* mRNA Expression on Gingival Tissue in Diabetic Mice After PD

**Figure 5:**
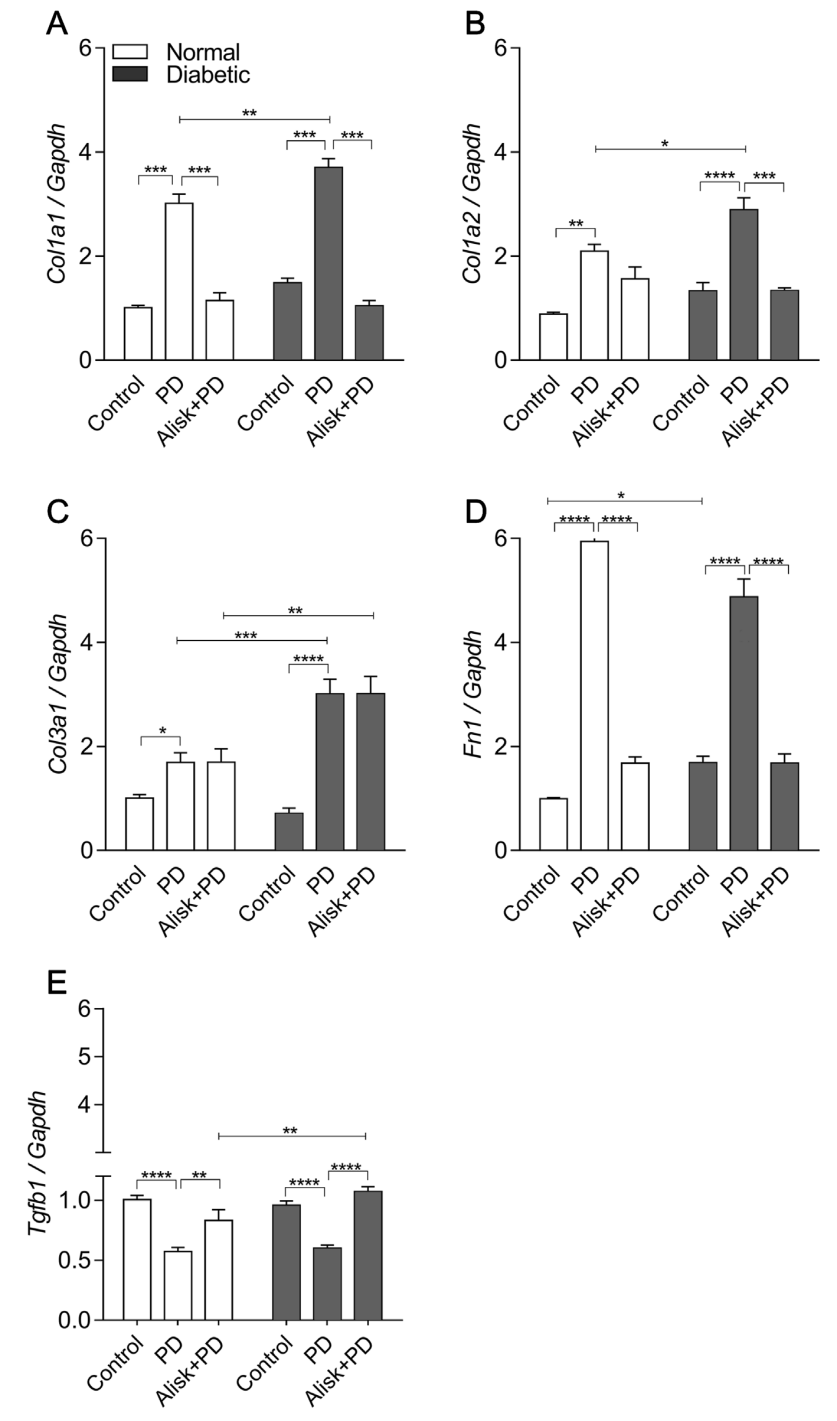
Col1a1, Col1a2, Col3a1, Fn1, and Tgfb1 gene expression of gingival tissue from normal and diabetic mice at 14 days after PD regardless of the fact if they were treated or not with aliskiren. **(A)** Col1a1, **(B)** Col1a2, **(C)** Col3a1, **(D)** Fn1, and **(E)** Tgfb1 in gingival tissue of control normal, normal with PD, normal with PD treated with aliskiren, control diabetic, diabetic with PD, and diabetic with PD treated with aliskiren by real-time RT-PCR. Results express mean ± SEM, normalized by fold change. Significant values are represented by p < 0.001, **p < 0.05, ***p < 0.005, and ****p < 0.0001 (Control vs. PD, PD vs. Alisk+PD, PD vs. PD, and PD+Alisk vs. PD+Alisk).

Aiming to accumulate evidence for modulation of renin in the tissue repair markers, their expression was evaluated in gingival tissue in normal and diabetic mice with PD, with or without treatment with Alisk. PD increased *Col1a1*, *Col1a2*, *Col3a1*, and *Fn* expression in normal and diabetic mice, when compared to respective control (**Figure 5A–E**). In normal mice, Alisk treatment reduced the expression of *Col1a1* and Fn1 (**Figure 5A, D, and E**), while in diabetic mice, Alisk reduced *Col1a1*, *Col1a2*, and *Fn* expression (**Figure 5B**). TGF-β was increased in the control animals, and PD induction caused a decrease in the mRNA expression. Alisk did not produce any effect on its levels (**Figure 5E**).

## Discussion

The results of our study demonstrated, for the first time, that renin causes exacerbation of inflammatory response and periodontal bone loss, primarily in diabetic mice. Furthermore, we confirmed the existence of components of local RAS in the gingival tissue of mice, as well as their involvement in the regulation of the components of extracellular matrix proteins after PD.

Renin is the rate-limiting enzyme of RAS, which can thereby stimulate the inflammation, vasopressor effects, and oxidative stress and induce fibrosis by cytokine released and chemokines mediating tissue inflammatory process (Campbell, 1987; Paul, 2006). Aliskiren is the first renin inhibitor to reach the market. It lowers elevated blood pressure efficiently by decreasing plasma and/or local renin activity (Jensen et al., 2008). The Alisk in the dose of 50 mg/kg improved bone alveolar loss and decreased inflammatory process. Corroborating with the literature, the dose used in the present study was also observed to decrease blood pressure, and renin–angiotensin II level in mice and attenuated steatosis and inflammation in mice (Lee et al., 2013; Wang et al., 2015; Chen et al., 2017). Furthermore, other study using the same dose of Alisk reduced levels of TNF-α and IL-6 in mice (Patel et al., 2013). IR for renin was found in gingival tissue of control normal mice but not in diabetic mice, but mRNA expression was undetectable. Although speculative, it is possible that circulating renin may be involved in the local process as a compensatory mechanism.

In the present study, renin-induced periodontal bone loss was observed in both animals; however, Alisk was able to reverse it more significantly in diabetic mice. Studies have demonstrated that AngII produced by bone cells is capable of stimulating osteoclastogenesis in MC3T3-E1 cells of calvaria (Hatton et al., 1997) and that the local RAS plays an important role in RANKL/OPG signaling modulating the bone metabolism of hypertensive and osteoporotic patients (Aoki et al., 2015; Shuai et al., 2015). Xue et al. (2016) demonstrated that renin inhibitor Alisk exhibited beneficial effects on trabecular bone of ovariectomy-induced osteoporotic mice. Thus, our results highlight, for the first time, the hypothesis that RAS might be contributing to the periodontal bone loss of diabetic mice after PD. The mechanism involved in this process is in progress for further study.

This study has also demonstrated the role of renin in the leukocyte recruitment during inflammatory process and/or infection. Alisk was able to prevent CRP production, which is an acute inflammatory protein that increases up to 1,000-fold at sites of infection or inflammation. Recently, there is growing evidence that CRP plays an important role in inflammatory processes and host responses to infection including the complement pathway, apoptosis, phagocytosis, nitric oxide release, and the production of cytokines, particularly IL-6 and TNF-α (Sproston and Ashworth, 2018). In our study, we observed elevated CRP production in the gingival tissue after 14 days of PD. Possible reasons for this can be, firstly, animals used in this study had PD suggesting presence of bacteria causing local inflammation. Antigens from bacteria can go into the circulation and activate hepatocytes; thereby, releasing CRP animals had PD, suggesting the presence of bacteria in the local of inflammation and antigens from bacteria going to the circulation and activating hepatocytes releasing CRP. Secondly, leukocytes recruited primarily by macrophages and lymphocytes can also be releasing local CRP (Sproston and Ashworth, 2018). Studies have confirmed CRP to be an important PD-induced gingival inflammation marker, which is already associated with leukocyte infiltrate (Bansal et al., 2014; Kumar et al., 2013), prothrombotic effects (Pasceri et al., 2000), and up regulation of AT1R (Wang et al., 2003). As an inference of our results, we suggest an important role of RAS in the PD-triggered inflammation (Li et al., 2011; Zhao et al., 2013). The inhibition of CRP by Alisk mainly in diabetic animals is possibly due to the capacity of this inhibitor to lead to decrease in the monocytes adhesion, inflammation, and oxidative stress (Pacurari et al., 2014).

A correct balance between neutrophils and monocytes/macrophages is crucial for the inflammation reduction and tissue repair attempts (Chapple et al., 1998; Cekici et al., 2014). It has already been shown that diabetes does not reduce leukocyte recruitment but impairs phagocytic ability of leukocytes and bacterial clearance (Pettersson et al., 2011). Thus, these differences in the chemokine production observed in the present study may represent one of the mechanisms responsible for an increased periodontal destruction in diabetics. The higher production of IL-1β in diabetic mice suggests that this cytokine is an important marker of severity of PD, which in presence of hyperglycemia induces activation of ACEs, causing local immune dysfunction and cytokine imbalance promoting elevation of IL-1β (Polak and Shapira, 2018). Alisk prevented increased production of CXCL2 in diabetics, and CCL8 in both animals. It suggests that, at least, renin is modulating CXCL2 and CCL8 production. Studies have already shown that AngII/Agtr1 axis is involved in neutrophil recruitment in different experimental models through CXC chemokines (Nabah et al., 2004; Shimada et al., 2011). Few studies relate RAS to CCL8, but an *in vitro* study has shown that AngII can stimulate CCL8 expression in human macrophage (Tone et al., 2007). *In vitro* studies have already shown that IL-1β attenuates renin expression *via* the p42/44 MAPK–STAT3 pathway (Liu et al., 2006). Our results show negative IR for renin production and expression in the gingival after 14 days of PD, which corroborates with previous results. Furthermore, elevated AngII alters systemic and local levels of pro- and anti-inflammatory cytokines such as IL-1β, IL-6, and TNF-α (Zhang et al., 2009). Conclusively, our data corroborates to literature, evidencing an important role of RAS in the cytokine/chemokine production, mainly in diabetic model. This helps in explaining the increased PD consequences and pronounces protective effects of Alisk on CXCL2 and CCL8. However, this has no effect on IL-β production possibly acting *via* a different regulatory mechanism; that is, it is not dependent on renin expression and/or production. Furthermore, IL-1β as an important pro-inflammatory cytokine is able to coordinate the fibroblast activation to produce other cytokines and/or chemokines as CXCL2 or CCL8 promoting a cascade mechanism (O’Hara et al., 2012; Gao et al., 2014; Alomar et al., 2015).

Aiming to confirm a possible correlation between RAS and PD in the gingival tissue, we further investigated the gene expression and protein production of RAS components in gingival tissue. Both qPCR and IHC experimental analyses confirmed the presence of RAS components in the normal and diabetic mice gingival tissue after 14 days of PD with different characteristics depending on the target evaluated.

Hepatic AGT is the major source of circulating AGT protein. However, the gingival tissue also produces local AGT as observed in our study. Regulation of *AGT* expression by cytokines has been shown in multiple organs and isolated immune cells (Brasier et al., 1990; Corvol et al., 1997; O’Leary et al., 2016). If this result can be applied to this study, then it seems reasonable to assume that CXCL2, CCL8, or IL-1β could be stimulating AGT production from gingival tissue after PD in both normal and diabetic mice. Therefore, PD-induced AGT production suggests that there is an increase in all angiotensin peptides, including AngII, which also plays a pro-inflammatory action. However, this needs further investigation. In the current study, Alisk significantly decreased *AGT* gene expression and protein production in both normal and diabetic mice. This is probably caused by reducing levels of renin and consequently circulating AngII, which, besides being a cytokine, regulates AGT production as has been already shown in a previous study (Kobori et al., 2001). Recent studies have shown that Alisk is effective in decreasing the progression of diabetes in db/db mice reducing proteinuria and renal inflammation and oxidative stress (Zhou et al., 2015).

In the present study, we found, for the first time, *ACE* expression and protein production in gingival tissue in both normal and diabetic mice after PD. The presence of ACE was also observed in human gingival fibroblasts, human periodontal ligament fibroblasts, and gingival tissue of rats with PD in serum, lung, heart, and liver of mice (Santos et al., 2015; Roca-Ho et al., 2017). Alisk was more effective in reducing ACE levels in diabetic mice to levels even below than what are seen in control group. This is possible as Alisk might be blocking the generation of AngI from AGT by causing renin inhibition, thereby reducing all angiotensin peptides. This might be promoting a down regulation of ACE due to feedback mechanism, as shown by previous other studies (Schunkert et al., 1992; Sadjadi et al., 2005). Information regarding regulation of ACE by inflammatory mediators is less; however, in an adjuvant induced arthritis model, the expression of *ACE* was found to be decreased in heart (Hanafy et al., 2011). For this reason, it is possible to speculate that the inflammatory mediators produced during PD in our model can be modulating ACE production.

The majority of ACE-derived angiotensin II actions mainly depend on their binding to their AT1R receptor, although there is also some affinity for the AT2R receptor. In the current study, for the first time, we found* AT1R* and *AT2R* expression and protein production in gingival tissue of normal and diabetic mice after PD. It has already been seen in normal rats with PD by Santos et al., 2009, 2015. AT1R and AT2R show similar properties of AngII binding but different genomic structure, localization, tissue-specific expression, and regulation (de Gasparo et al., 2000). Although it is speculative, the higher *AT1R* and *AT2R* expression and protein production seen in the gingival tissue of normal and diabetic mice after PD can be happening because of increased cytokine and chemokine release by gingival fibroblast, thereby increasing AngII production by acting on their specific receptors (Forrester et al., 2018; Sato et al., 2018). Furthermore, the exacerbated consequences of PD in diabetic animals, which present higher AT1R response, can be related to bone loss and inflammation promotion, combined to a higher AT2R response, which shows protective effects in inflammation and tissue injury (Benndorf et al., 2009; Abadir et al., 2011; Dhande et al., 2015). This suggests an altered endogenous mechanism in the diabetic condition. Renin also regulates these AngII receptors, as Alisk was able to significantly reduce AT1R and AT2R in both normal and diabetic mice. Studies with clinical screening have shown that the use of inhibitors of RAS components reduces the incidence of cardiovascular complications and inflammation in diabetic patients (Lewis et al., 2001). Taken together, these data suggest that local and/or systemic renin can be involved in the expression and production of AT1R and AT2R. Alisk presented a protective effect in the gingival tissue, possibly by reducing AngII levels and, consequently, the inflammatory process that is responsible to trigger AT1R activation and hence altering the local RAS in periodontitis.

In the current study, we demonstrated for the first time mRNA expression and protein production of ACE2 and MasR in gingival tissue of normal and diabetic mice after 14 days of PD. ACE2 is able to convert AngII into Ang1–7, which acts through MasR. The activation of this receptor, in turn, shows opposite effect to AngII/AT1R axis, comprising an important endogenous mechanism that eliminates AngII produced and counteracts its effects (Rodrigues Prestes et al., 2017). Our results showed high expression of *ACE2* in both animals. However, in diabetic mice, this expression was lower as compared to normal mice. These results are in agreement with previous studies that have also observed that *ACE2* was reduced in streptozotocin-induced diabetic animals regulating the tissue and plasma level of AngII, preventing development of cardiovascular disease (Tikellis et al., 2008; Tikellis et al., 2012). PD decreased *ACE2* expression in normal mice, but not in diabetic mice. This suggests that, although speculative, cytokines released during the local gingival inflammatory process were able to inhibit the production of ACE2 primarily in normal mice, as ACE2 can be reduced in diabetic mice. Studies showed that the activation of the ACE2/Ang-(1–7)/MasR axis can modulate the expression of pro-inﬂammatory cytokines in a model of pulmonary hypertension. Indeed, there was decreased expression of *TNF-α*, *IL-1β*, *IL-6*, *MCP-1*, and *TGF-β* and increased expression of the anti-inﬂammatory cytokine *IL-10* (Ferreira et al., 2009; Yamazato et al., 2009; Shenoy et al., 2010). In the present study, the inflammatory response provoked by PD in diabetic mice was more pronounced when compared to normal mice. For this reason, it is possible that some imbalance occurs in the ACE2/Ang1–7 axis promoting a poor prognosis in diabetic mice with PD. Alisk decreased the *ACE2* mRNA expression and protein production in diabetic mice, but not in normal mice, suggesting that renin production can also be involved in this modulation. Studies have demonstrated that the pathogenesis of diabetes is mediated by an upregulation of ACE and a downregulation of ACE2, suggesting a compensatory mechanism (Mizuiri et al., 2008).

In the current study, although speculative, it is possible that AT2R can be modulating the MasR, since, when *AT2R* expression is decreased, *MasR* expression is increased in control group. In the presence of PD, *AT2R* has a huge enhancement in expression possibly because MasR remained equal to control group. Previous studies conducted in the rat stroke model point out presence of AngII/AT2R signaling in the brain, which enhances Ang-(1–7)/MasR, antagonizing repressor response after stroke (Chang and Wei, 2015). Additionally, Patel et al. (2017), using obese rat kidney, observed that AT2R and MasR can colocalize and are functionally interdependent in terms of stimulation by nitric oxide (NO). In the present study, we observed that renin is involved in the modulation of MasR, more effectively in diabetic mice. Studies showed that the novel renin inhibitor Alisk antagonizes the stroke-induced repressor response by reducing AngII, Ang-(1–7), and AngIV levels (Chang and Wei, 2015). Corroborating with the present study, our data showed that AT1R, AT2R, ACE2, and MasR were decreased after Alisk treatment, suggesting the reduction in their response.

The RAS operates predominantly through AngII, which is a major regulator of ﬁbroblast homeostasis (Brilla, 2000). Type 1 collagen and type 3 collagen have already been related to PD progression and other inflammatory conditions (Lorencini et al., 2009). Since gingival tissue has lots of fibroblast producing ECM, it is of utmost importance to our study, whereas after PD, the collagen production was increased. Studies have already reported increased collagen expression in PD model as a compensatory mechanism and repair effort due to increased matrix degradation (Larjava et al., 1989; Larjava et al., 1990). AngII has already been shown to stimulate collagen synthesis in cardiac fibroblasts (Lijnen et al., 2001) and diabetic skin fibroblasts (Ren et al., 2013). Besides that, AngII leads to dose-dependent collagen production within the myocardium and has emerged as a key mediator of myocardial ﬁbrosis (Brilla et al., 1994). However, studies have demonstrated that AT1R inhibitor, but not AT2R inhibitor, was able to decrease Col 1, Col 3, and TGF-β in diabetic skin fibroblasts, suggesting that this effect is mediated through AT1R stimulation independent of AT2R (Ren et al., 2013). In this study, Alisk reduced *Col1a1* expression, in both animals, and *Col1a2* expression only in diabetic mice, suggesting that both are regulated by renin. On the other hand, *Col3a1* expression was not altered, suggesting that it is not regulated by renin mechanism. *Fn1* expression, also an extracellular matrix glycoprotein related to tissue repair and cell adhesion/migration (Ren et al., 2013), was found to be significantly increased after PD in normal and diabetic mice, suggesting that its production is mediated by renin release. Recent studies have already shown that AngII stimulates Fn synthesis in proximal tubular (Alexander et al., 2014) and collecting duct cells (Cuevas et al., 2015) by different pathway. An important point is that wound healing process occurs in three phases: inflammation, proliferation, and remodeling. However, some abnormality and imbalance in the production and destruction of collagens can lead to formation of hypertrophic scars and keloids (Mari et al., 2015). In one study, patients with hypertrophic scars and keloids were treated with AngII receptor antagonist and ACE inhibitors and they were effective to inhibit them (Niazi et al., 2018). For these reasons, it is possible to speculate that Alisk can be used as a pharmacological tool to inhibit the excess of Col1 and Fn1 production during the PD. Further investigation needs to be conducted to warrant and support this.

In the present study, PD significantly reduced *TGF-β1* expression in both animal groups. Previous studies have demonstrated that although TGF-β1 levels are elevated in moderate disease, progression of PD can decrease this response in fluid samples obtained from the periodontal pockets (Skalerič et al., 1997). Then, this can explain the reduction of TGF-β in gingival tissue of animals after 14 days. Thus, ambiguous role of TGF-β1 in periodontal wound healing remains unclear. Studies have shown that concentration of TGF-β1 in the gingival tissue exhibits dynamic changes associated with the progression of experimental periodontal inflammation (Skalerič et al., 1997; Ko et al., 1999; Shah and Hux, 2003; Muller et al., 2005; Gürkan et al., 2006; Ribeiro-Oliveira et al., 2008). The levels of TGF-β1 in gingival tissue may be valuable to detect the inflammatory reaction in the periodontal tissue. In addition, Alisk was able to reverse this response, increasing these levels mainly in diabetic mice. These results suggest that *TGF-β1* expression is dependent on renin and possibly on its derivatives such as AngII-dependent mechanisms, being able to participate in this response. Further studies are necessary to elucidate the relationship between RAS and TGF-β production in PD progression.

In summary, the results suggest that local RAS in gingival tissue not only does exist but also is functional in normal and diabetic mice in periodontal tissue, modulating the inflammatory and wound healing process. Furthermore, inhibiting the renin enzymatic activity can improve periodontal bone loss and inflammatory response and exacerbate wound healing process, mainly in diabetes. Renin inhibitor can be a possible tool to prevent bone and tissue implication during the PD during the diabetes.

## Data Availability Statement

The raw data supporting the conclusions of this manuscript will be made available by the authors, without undue reservation, to any qualified researcher.

## Ethics Statement

We certify that the study entitled “Renin–angiotensin system and periodontal bone metabolism: involvement of microRNAs and mast cells in inflammatory response in animal models of hypertension and diabetes,” Protocol FOA no. 00974-2016, under the supervision of Sandra Helena Penha de Oliveira presents an experimental protocol in accordance with the Ethical Principles of Animal Experimentation and its implementation was approved by CEUA on April 19, 2017.

## Author Contributions

SO conceived and designed the experiments. VB, SF, BR, MF, DQ, and CB performed the experiments. SO, VB, SF, and DQ analyzed the data. SO, CS, and VL contributed reagents/materials/analysis tools. SO, VB, and DQ wrote the paper.

## Funding

This work was supported by two types of research grants as follows: The São Paulo Research Foundation [FAPESP-2015/03965-2 (SHPO, CFS, VSL), 2018/04989-0 (MNF), 2018/04476-3 (BSR), 2017/05873-3 (SCTF)] and the Coordination for the Improvement of Higher Education Personnel (CAPES) (VGBB, DPQ, CTB).

## Conflict of Interest Statement

The authors declare that the research was conducted in the absence of any commercial or financial relationships that could be construed as a potential conflict of interest.
